# Health Tract, No. 80

**Published:** 1862-07

**Authors:** 


HEALTH TRACT, No. 80.

### SCALDS AND BURNS.

On the instant of the accident, plunge the part under cold water. This relieves the pain in a second, and allows all hands to become composed. If the part can not be kept under water, cover it over with dry flour, an inch deep or more. In both cases pain ceases because the air is excluded. In many instances nothing more will be needed after the flour; simply let it remain until it falls off, when a new skin will be found under. In severer cases, while the part injured is under water, simmer a leek or two in an earthen vessel, with half their bulk of hog's lard, until the leeks are soft, then strain through a muslin rag. This makes a greenish-colored ointment, which, when cool, spread thickly on a *linen* cloth and apply it to the injured part. If there are blisters, let out the water. When the part becomes feverish and uncomfortable, renew the ointment, and a rapid, painless cure will be the result, if the patient, in the mean while, lives exclusively on fruits, coarse bread, and other light, loosening food.

If the scald or burn is not very severe—that is, if it is not deeper than the outer skin—an ointment made of sulphur, with lard enough to make it spread stiffly on a linen rag, will be effectual. The leek-ointment is most needed when there is ulceration from neglected burns, or when the injury is deeper than the surface. As this ointment is very healing and soothing in the troublesome excoriations of children, and also in foul, indolent ulcers, and is said to be efficacious in modifying, or preventing altogether, the pitting of small-pox, it would answer a good purpose if families were to keep it on hand for emergencies—the sulphur-ointment for moderate cases, and the leek-ointment in those of greater severity, or of a deeper nature.

